# Facilitating standardized COVID-19 suspicion prediction based on computed tomography radiomics in a multi-demographic setting

**DOI:** 10.1007/s00330-022-08730-6

**Published:** 2022-04-01

**Authors:** Yeshaswini Nagaraj, Gonda de Jonge, Anna Andreychenko, Gabriele Presti, Matthias A. Fink, Nikolay Pavlov, Carlo C. Quattrocchi, Sergey Morozov, Raymond Veldhuis, Matthijs Oudkerk, Peter M. A. van Ooijen

**Affiliations:** 1 Department of Radiation Oncology, University of Groningen, University Medical Center Groningen, Hanzeplein 1, 9713 GZ Groningen, The Netherlands; 2 Machine Learning Lab, DASH, University of Groningen, University Medical Center Groningen, Groningen, The Netherlands; 3 Department of Radiology, University of Groningen, University Medical Center Groningen, Groningen, The Netherlands; 4 Research and Practical Clinical Center for Diagnostics and Telemedicine Technologies of the Moscow Healthcare Department, Moscow, Russia; 5 Unit of Diagnostic Imaging and Interventional Radiology, Departmental Faculty of Medicine and Surgery, Università Campus Bio-Medico di Roma, Rome, Italy; 6 Clinic for Diagnostic and Interventional Radiology, University Hospital Heidelberg, Heidelberg, Germany; 7 Translational Lung Research Center Heidelberg, member of the German Center for Lung Research, Heidelberg, Germany; 8 Faculty of Electrical Engineering, Mathematics Computer Science (EWI), Data management Biometrics (DMB), University of Twente, Enschede, The Netherlands; 9 Faculty of Medical Sciences, University of Groningen, Groningen, The Netherlands; 10 Institute for DiagNostic Accuracy Research, Groningen, The Netherlands

**Keywords:** COVID-19, Deep learning, Diagnostic imaging, SARS-CoV-2, Tomography X-ray computed

## Abstract

**Objective:**

To develop an automatic COVID-19 Reporting and Data System (CO-RADS)–based classification in a multi-demographic setting.

**Methods:**

This multi-institutional review boards–approved retrospective study included 2720 chest CT scans (mean age, 58 years [range 18–100 years]) from Italian and Russian patients. Three board-certified radiologists from three countries assessed randomly selected subcohorts from each population and provided CO-RADS–based annotations. CT radiomic features were extracted from the selected subcohorts after preprocessing steps like lung lobe segmentation and automatic noise reduction. We compared three machine learning models, logistic regression (LR), multilayer perceptron (MLP), and random forest (RF) for the automated CO-RADS classification. Model evaluation was carried out in two scenarios, first, training on a mixed multi-demographic subcohort and testing on an independent hold-out dataset. In the second scenario, training was done on a single demography and externally validated on the other demography.

**Results:**

The overall inter-observer agreement for the CO-RADS scoring between the radiologists was substantial (*k* = 0.80). Irrespective of the type of validation test scenario, suspected COVID-19 CT scans were identified with an accuracy of 84%. SHapley Additive exPlanations (SHAP) interpretation showed that the “wavelet_(LH)_GLCM_Imc1” feature had a positive impact on COVID prediction both with and without noise reduction. The application of noise reduction improved the overall performance between the classifiers for all types.

**Conclusion:**

Using an automated model based on the COVID-19 Reporting and Data System (CO-RADS), we achieved clinically acceptable performance in a multi-demographic setting. This approach can serve as a standardized tool for automated COVID-19 assessment.

**Keypoints:**

*• Automatic CO-RADS scoring of large-scale multi-demographic chest CTs with mean AUC of 0.93 ± 0.04.*

*• Validation procedure resembles TRIPOD 2b and 3 categories, enhancing the quality of experimental design to test the cross-dataset domain shift between institutions aiding clinical integration.*

*• Identification of COVID-19 pneumonia in the presence of community-acquired pneumonia and other comorbidities with an AUC of 0.92.*

**Supplementary Information:**

The online version contains supplementary material available at 10.1007/s00330-022-08730-6.

## Introduction

Previously published articles have shown that COVID-19 has distinct imaging features making screening of suspected cases and evaluating disease progression using CT possible [1, 2]. Based on CT findings, several radiological societies have released standardized protocols for suspicion staging COVID-19 patients [3–5]. The COVID-19 Reporting and Data System or CO-RADS is a five-class suspicion classification scheme released by the Dutch Radiological Society (NVvR) [4]. CO-RADS includes discerning features related to unequivocal noninfectious origins, or community-acquired pneumonia from typical COVID-19 features on an unenhanced chest CT in a population with high incidence of COVID-19. This reporting system has shown high discriminatory power in triaging COVID-19 and provides an appropriate reporting language understandable by any radiologist [6, 7]. Therefore, using a standardized score such as CO-RADS is the most straightforward way of implementing an automated COVID-19 detection method [8].

The urgency to aid radiologists to detect COVID-19 has resulted in a rush to develop machine learning and deep learning models by neglecting a standardized approach. For example, the use of non-generalizable data for model training (single demographic, public datasets with different acquisition or reconstruction protocols, unavailability of source DICOM, scans with image artifacts) has resulted in poor application of machine learning methodology. This limitation has led to reproducibility issues and biases in recent study designs [9]. This can further aggravate as data characteristics change based on demographics, immunity landscape and clinical practice shift between different pandemic stages. Therefore, AI-driven studies should follow standardized and reproducible pathways to confirm the performance of AI models and their rapid adaptability and implementation into the clinical workflows.

Radiomics is a method of quantifying phenotypic characteristics of lesions in medical imaging using mathematical algorithms which can then be used to predict disease severity and progression [10, 11]. This quantitative process contrasts with the conventional radiological method, where the radiologist describes the lesions mainly based on qualitative attributes. The radiomic features are extracted at the sub-visual level, meaning that the computer system can detect patterns that might not be discernible by the human visual system. Therefore, when used in a standardized environment, radiomics may provide valuable clinical information complementary to conventional radiological analysis [12].

For the use of automated models in clinical practice, it is essential to consider three key aspects of model validation. The first aspect is to acquire high-quality data from a diverse population (multi-demographic) cohort. The second is to adopt a standardized annotation protocol understandable by radiologists. The third is to perform a thorough model analysis using various testing scenarios (internal–external validation). In our study, apart from including the aforementioned aspects, we performed model evaluation using datasets processed with and without noise reduction and interpreted the model output using radiomic features based on SHapley Additive exPlanations (SHAP) [13]. We hypothesize that in a multi-demographic setting, COVID-19 can be discerned automatically by predicting the CO-RADS score on chest CT using a classification algorithm combined with an optimal radiomic signature.

## Materials and methods

**Fig. 1:**
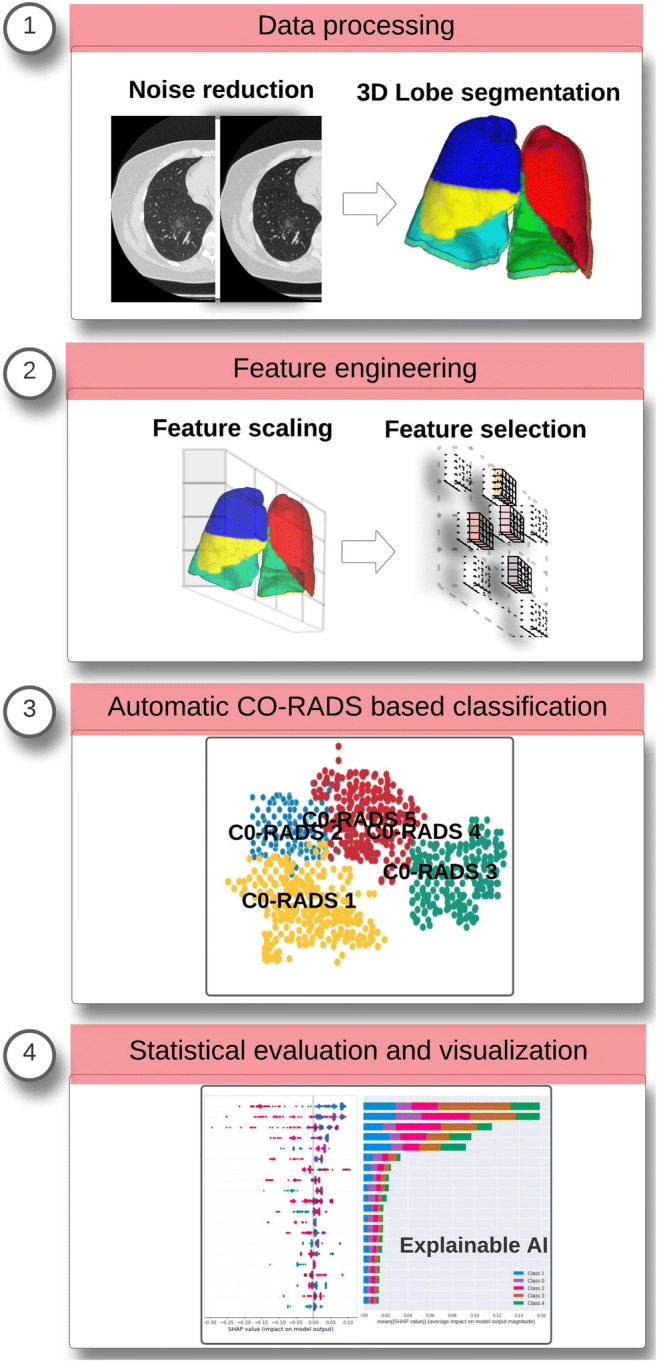
The overall workflow followed to validate machine learning models for automated COVID-19 suspicion staging based on the CO-RADS protocol. The scans with COVID-19 suspicion were selected from the Italian and Russian subcohorts retrospectively and annotated by experienced radiologists from different countries. In the first step, the datasets were processed using deep learning–based noise reduction (DLNR) and 3D segmentation masks were generated for each scan. Next, radiomic features were extracted and classified using ML algorithms. In the final step, statistical evaluation of the standard performance metrics and visualization of class-specific features were carried out to enhance the explainability of the models

This retrospective study was approved by the ethics committees of the participating institutions. A graphical abstract of the workflow is shown in Fig. 1.

### Study population

**Fig. 2:**
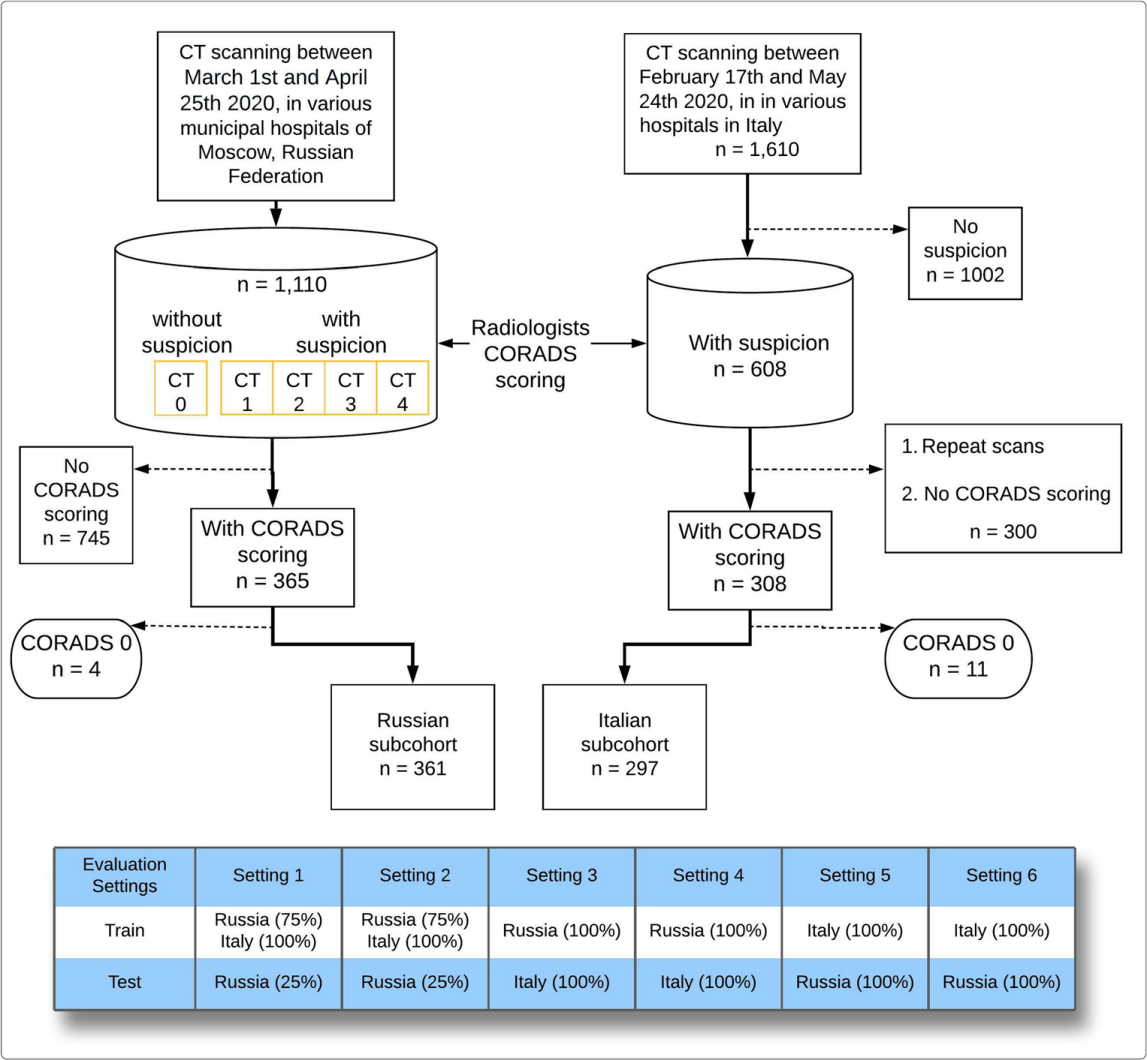
Data flowchart of Russian and Italian subcohorts included for the study with the training and test split. Below the flowchart, a description of different evaluation settings of data scenarios is depicted. Note that *n* refers to the number of patients. CO-RADS–COVID-19 Reporting and Data Systems, CT 0, 1, 2, 3, 4–severity–based Russian annotations

This study included 1418 chest CTs from patients suspected of having COVID-19 from two countries: the first population, from MosMed in Moscow, Russia, collected between March and April 2020 and the second population from the COVID center of Università Campus Bio-Medico di Roma, Rome, Italy, collected between February and May 2020 [14, 15]. Although the acquisition protocol was entirely different for both populations, the received data set primarily consisted of anonymized unenhanced chest CT imaging with an average of 300–400 images (slices) per patient scan. The inclusion and exclusion criteria of the acquired dataset are shown in Fig. 2, and the overview of the acquisition and reconstruction parameters is shown in Table 1.

**Table 1:** Acquisition and reconstruction parameters of Italian and Russian subcohorts

	Italy		Russia	
Acquisition parameters				
Scanner	SOMATOM force		Toshiba Aquilion64	
Scan mode	Spiral		Helical	
Pitch	1.2–1.5		1.484	
Tube voltage [kVp]	100–150		120	
Tube current [mAs]	33–296		80–500 (automatically adjusted to achieve noise level of 10 HU for 5.0-mm-thick slices)	
Contrast	No		No	
API (inspiration/expiration/mixed)	Inspiration		Suspended inspiration	
Direction	Craniocaudal		Caudocranial	
Upper limit	Pulmonary apex		5 cm above lungs	
Lower limit	Lower diaphragmatic limit		5 cm below lungs	
Reconstruction parameters				
Slice thickness [mm]	1–3		1.0	
Slice increment [mm]	1		0.8	
FOV [mm]	350–500		350–500	
Reconstruction kernel	BL64–BR40		FC07	
Reconstruction method	Iterative		QDS + (FBP)	
Window width [HU]	300/400	1500/1600	400	1500
Window center [HU]	30/40	− 500/− 600	40	− 500

### Radiologists’ annotation

To explicate the acquired datasets, three board-certified multi-demographic radiologists (Electronic supplementary material, section 1) with more than 5 years of experience reviewed the CT images from multi-demographic datasets. The CO-RADS annotation protocol was followed by each radiologist to capture the multi-class variation in COVID-19 suspected candidates. The readers were blinded to the clinical–epidemiological diagnosis of COVID-19 and the predefined CT category (severity levels) that existed in the Russian dataset. In the given time, we were able to retrieve systematic annotations for 673 patient scans. An overlap subset of 43 patients was selected randomly from the included population to calculate the inter-observer agreement between multi-demographic radiologists. The CT scans were viewed using an open-source Multi-image Analysis GUI [16], with readers being able to modify the window settings.

### Noise reduction

In our study, we used a proprietary deep learning–based noise reduction (DLNR) algorithm (Pixelshine, AlgoMedica) which has been shown to effectively reduce image noise of an already reconstructed DICOM image and can improve the image quality [17–19]. All the scans irrespective of CT protocol were processed using the DLNR algorithm for analysis. In total, two subsets of data were created, one without noise reduction (setting 1, 3, and 5) and one with noise reduction (setting 2, 4, and 6). The reason for using noise reduction is to ensure a more consistent presentation of the data obtained with different protocols.

### Feature extraction

After 3D lung lobe segmentation (Electronic supplementary material, section 2) of each scan, radiomic features were derived using the PyRadiomics library [20]. Most of the feature descriptors extracted using PyRadiomics are in compliance with feature definitions described by the Imaging Biomarker Standardization Initiative (IBSI) [21]. Additionally, 26 new radiomic descriptors known as co-occurrence of local anisotropic gradient orientations (CoLlAGe) features were included to capture the lobe-level anisotropy and encapsulate CO-RADS [22].

Furthermore, to increase the feature diversity, features were extracted after applying filters such as Wavelet, Laplacian of Gaussian (LoG), Square, Square Root, Logarithmic, Exponential, Gradient, and Local Binary Pattern 2D (LBP2D) to the original image. However, filters were not applied to the two-dimensional shape–based category and CoLlAGe features.

### Experimental design and performance evaluation

We used the machine learning (ML) models logistic regression (LR), multilayer perceptron (MLP), and random forest (RF) for automated CO-RADS classification. For comparison purposes, we evaluated the ML models under different combinations of CO-RADS operating points as described recently [8]. To account for the generalizability and reproducibility using multi-demographic datasets in the three machine learning models, we considered two scenarios for model evaluation. In the first scenario, we trained the classification models on a mixed dataset (Russian and Italian datasets) and tested on an internal hold-out dataset (Russian). In the second scenario, the classification models were trained either on the Russian or on the Italian dataset and externally validated on the other population. Since these scenarios were evaluated with and without noise reduction for classification, in total, we implemented six different settings to thoroughly evaluate all the possibilities of model training. The details of each setting including the data split are shown in Fig. 2.

### Statistics and evaluation metrics

To understand the inter-observer variability in CO-RADS annotation between the radiologists, we used Fleiss kappa and *t* test.

Before model training, the extracted features were scaled to the same bandwidth using *Z*-score normalization. We used the Pearson’s correlation score (PCC), to eliminate the redundant features with PCC greater than 0.95. As a feature selection method, we used one-way ANOVA *F*-test and features with a significant difference (*p* < 0.05) between the groups were considered [23]. This feature scaling and feature selection application is referred to as feature engineering in the rest of the paper. To avoid over-fitting due to class imbalance that existed in CO-RADS classes, we used the Synthetic Minority Oversampling Technique (SMOTE) [24].

In our experiments, we calculated the area under the receiver operation curve (AUC) to understand the performance of ML–based automatic CO-RADS scoring along with F1 measure, recall, accuracy, and precision. DeLong’s test was used to compare the statistical difference between AUC [25]. The 95% confidence interval (95% CI) for the metrics was evaluated using non-parametric bootstrapping with 1000 iterations. The complete experimental setup was built in-house using the PyTorch framework in Python [26].

### Explainable visualizations

For better understanding of model prediction, we used SHapley Additive exPlanations (SHAP) which measures the feature importance based on the model output [27]. The SHAP five-class plot was used to visualize feature importance on the CO-RADS classification for the best model. The independent class summary plot for COVID-19 (CO-RADS 5) was used to understand the relationship between the feature and the impact on the prediction.

## Results

### Patient characteristics

We excluded 15 scans for which CO-RADS grade 0 (not interpretable) was assigned. In total, 34.5% (227/658) of the patients had no COVID-19 CT findings (CO-RADS 1) or normal, and 6.8% (45/658) of the patients had CT findings consistent with infections other than COVID-19 (CO-RADS 2), which mainly included multiple nodules, emphysema, fibrosis, bronchiectasis, calcification, lung abscess, tree-in-bud patterns, and pulmonary metastasis. About 17.2% (113/658) of the patients who had CT findings compatible with COVID-19, but also other disease like bacterial infection (aspecific GGOs, consolidations, pleural fluids), were categorized as CO-RADS 3. The CO-RADS 4 category contained 16.6% (109/658) of the patients who were suspected with COVID-19 abnormalities, and 24.9% (164/658) of the patients who had confirmed COVID-19 infection were categorized as CO-RADS 5 (Table 2). In the confirmed cases of both the subcohorts, more than 50% (194/297, 200/361) of the cases were found to have ground glass opacities. Other commonly observed findings were pleural effusion, consolidation, crazy paving, and vascular thickening.

**Table 2:** Data characteristics and CT features of participants in Italian and Russian subcohorts

Parameter	Italy	Russia	Total
*n*	308	365	673
Age	–	18–97 years	–
No. of CT scans per scoring			
CO-RADS 1	87 (29%)	140 (38%)	227
CO-RADS 2	15 (5%)	30 (8%)	45
CO-RADS 3	74 (25%)	39 (11%)	113
CO-RADS 4	45 (15%)	64 (18%)	109
CO-RADS 5	76 (26%)	88 (24%)	164
CT patterns			
GGO	194 (65%)	200 (55%)	394
Pleural effusion	77 (26%)	3(0.8%)	80
Consolidation	101 (34%)	102(28%)	203
Crazy paving	74 (25%)	30(8%)	104
Vascular thickening	63 (21%)	106(29%)	169

Number of samples (*n*) includes CO-RADS-0

### Inter-observer agreement of multi-demographic radiologist CO-RADS grading systems

For overall CO-RADS annotation, we observed substantial agreement between the radiologists with a mean Fleiss’ kappa (*k*) of 0.802 (95% CI: 0.705–0.899). For individual CO-RADS class, between the readers, there was almost perfect agreement for CO-RADS 5 (*k* = 1.00, 95% CI: 0.84–1.17), CO-RADS 1 (*k* = 0.93, 95% CI: 0.76–1.10), and CO-RADS 3 (*k* = 0.91, 95% CI: 0.74–1.00). Substantial agreement was observed for CO-RADS 2 and CO-RADS 4 with a *k* value of 0.76 (95% CI: 0.58–0.93) and 0.72 (95% CI: 0.56–0.92) respectively (Table 3).

**Table 3:** Inter-observer variability between radiologists for each CO-RADS classification

Type	Kappa	(95% CI) Lower	(95% CI) Upper	*p* value
Overall	0.868	0.705	1.20	< 0.001
CO-RADS 1	0.938	0.765	1.10	< 0.001
CO-RADS 2	0.769	0.589	0.934	< 0.001
CO-RADS 3	0.913	0.741	1.00	< 0.001
CO-RADS 4	0.721	0.560	0.92	< 0.001
CO-RADS 5	1.00	0.841	1.17	< 0.001

*CI* confidence interval

### Performance of models in different data settings

In this section, we report mean AUC of each classifier for overall CO-RADS classification and the percentage change in performance after noise reduction.

The logistic regression (LR) classifier, in setting 1, performed with a mean AUC of 0.83 ± 0.01 for overall CO-RADS classification and 0.83 ± 0.025 after noise reduction (setting 2). In settings 4 and 6 (after noise reduction), the LR classifier performance increased by 4% when compared to settings 3 and 5; that is, AUC increased from 0.71 ± 0.06 to 0.74 ± 0.04 and from 0.70 ± 0.02 to 0.75 ± 0.03 respectively.

The multilayer perceptron (MLP) classifier in setting 1 and setting 2 performed with an AUC of 0.84 ± 0.02 and 0.87 ± 0.06 respectively. In setting 3, the AUC was 0.72 ± 0.02, and in setting 4, the AUC was 0.77 ± 0.04. In setting 4, the AUC was 0.69 ± 0.021, and in setting 5, the AUC was 0.75 ± 0.01. Hence, after noise reduction, we observed 3%, 5%, and 6% increase in the performance of the MLP classifier in settings 2, 4, and 6 respectively.

The random forest model had an AUC of 0.88 ± 0.02 in setting 1 and 0.93 ± 0.04 in setting 2. In setting 3, the AUC was 0.76 ± 0.04, and in setting 4, the AUC was 0.78 ± 0.04. In setting 5, the AUC was 0.70 ± 0.02, and in setting 6, the AUC was 0.77 ± 0.02. Hence, after noise reduction, we observed 5%, 2%, and 7% increase in performance settings 2, 4, and 6 respectively. RF classifier outperformed other classifiers in most of the settings.

**Fig. 3:**
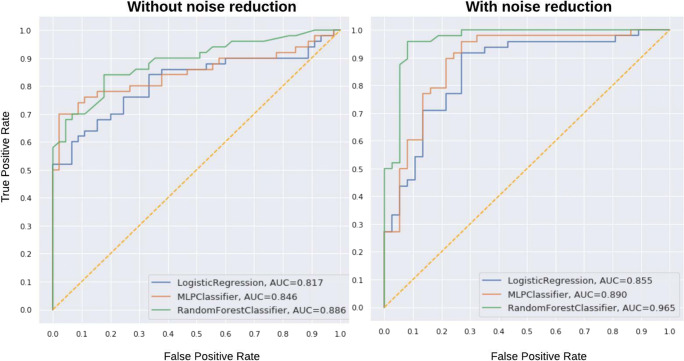
The receiver operating curves of machine learning algorithms showing their ability to classify normal chest CT from other CO-RADS stages on the hold-out dataset (scenario 1). The performance of the classifiers increased after noise reduction

The details of the CO-RADS score of all combinations for COVID predictions are shown in Table 4, and the other evaluation metrics are shown in Table 5. It can be observed that the performance of all the three classifiers in settings 1 and 2 was better than that of the rest of the settings. Additionally, we investigated the model capability for prediction of “normal chest CT” both with and without noise reduction and the plot is shown in Fig. 3. Among all the three ML classifiers, random forest performed the best in almost all settings.

**Table 4:** Area under the receiver operating curve (AUC) of all the machine learning (ML) algorithms for respective CO-RADS classification in each setting

Settings	Type	Logistic regression	Multilayer perceptron	Random forest
Setting 1	CO-RADS ≥ 2	0.82 (± 0.08)	0.85 (± 0.06)	0.89 (± 0.07)
	CO-RADS 3 + 4 + 5	0.83 (± 0.06)	0.85 (± 0.06)	0.89 (± 0.06)
	CO-RADS 4 + 5	0.85 (± 0.06)	0.86 (± 0.06)	0.84 (± 0.06)
	CO-RADS 5	0.82 (± 0.08)	0.81 (± 0.07)	0.88 (± 0.06)
Setting 2	CO-RADS ≥ 2	0.86 (± 0.06)	0.89 (± 0.06)	0.97 (± 0.04)
	CO-RADS 3 + 4 + 5	0.83 (± 0.06)	0.92 (± 0.06)	0.94 (± 0.06)
	CO-RADS 4 + 5	0.84 (± 0.06)	0.88 (± 0.05)	0.88 (± 0.04)
	CO-RADS 5	0.80 (± 0.04)	0.79 (± 0.04)	0.92 (± 0.07)
Setting 3	CO-RADS ≥ 2	0.77 (± 0.09)	0.75 (± 0.07)	0.79 (± 0.08)
	CO-RADS 3 + 4 + 5	0.75 (± 0.06)	0.71 (± 0.06)	0.73 (± 0.06)
	CO-RADS 4 + 5	0.65 (± 0.06)	0.70 (± 0.07)	0.79 (± 0.09)
	CO-RADS 5	0.66 (± 0.06)	(0.71 ± 0.06)	0.71 (± 0.06)
Setting 4	CO-RADS ≥ 2	0.78 (± 0.08)	0.82 (± 0.06)	0.83 (± 0.07)
	CO-RADS 3 + 4 + 5	0.77 (± 0.06)	0.78 (± 0.05)	0.78 (± 0.03)
	CO-RADS 4 + 5	0.73 (± 0.06)	0.73 (± 0.06)	0.75 (± 0.05)
	CO-RADS 5	0.70 (± 0.06)	0.73 (± 0.06)	0.75 (± 0.04)
Setting 5	CO-RADS ≥ 2	0.69 (± 0.06)	0.67 (± 0.06)	0.68 (± 0.06)
	CO-RADS 3 + 4 + 5	0.73 (± 0.08)	0.72 (± 0.07)	0.73 (± 0.06)
	CO-RADS 4 + 5	0.70 (± 0.06)	0.68 (± 0.07)	0.69 (± 0.07)
	CO-RADS 5	0.70 (± 0.07)	0.69 (± 0.10)	0.71 (± 0.08)
Setting 6	CO-RADS ≥ 2	0.74 (± 0.06)	0.76 (± 0.06)	0.76 (± 0.06)
	CO-RADS 3 + 4 + 5	0.71 (± 0.04)	0.76 (± 0.06)	0.77 (± 0.07)
	CO-RADS 4 + 5	0.79 (± 0.06)	0.73 (± 0.08)	0.79 (± 0.07)
	CO-RADS 5	0.74 (± 0.09)	0.75 (± 0.09)	0.74 (± 0.08)

**Table 5:** Performance metrics of the best machine learning (ML) algorithms for respective CO-RADS classification in each setting

Settings	Type	TP	FP	Precision	Recall	*F*-measure	AUC
Setting 1	CO-RADS ≥ 2	50	5	0.91	0.91	0.91	0.89 (± 0.07)
	CO-RADS 3 + 4 + 5	44	7	0.86	0.92	0.89	0.89 (± 0.06)
	CO-RADS 4 + 5	35	9	0.80	0.92	0.85	0.86 (± 0.06)
	CO-RADS 5	19	8	0.70	0.86	0.78	0.88 (± 0.06)
Setting 2	CO-RADS ≥ 2	52	2	0.96	0.95	0.95	0.97 (± 0.04)
	CO-RADS 3 + 4 + 5	45	5	0.90	0.94	0.92	0.94 (± 0.06)
	CO-RADS 4 + 5	36	9	0.80	0.95	0.87	0.88 (± 0.06)
	CO-RADS 5	20	4	0.83	0.91	0.87	0.92 (± 0.07)
Setting 3	CO-RADS ≥ 2	159	12	0.93	0.76	0.83	0.79 (± 0.07)
	CO-RADS 3 + 4 + 5	124	15	0.89	0.64	0.74	0.75 (± 0.06)
	CO-RADS 4 + 5	100	52	0.66	0.83	0.73	0.79 (± 0.09)
	CO-RADS 5	55	72	0.43	0.72	0.54	0.71 (± 0.06)
Setting 4	CO-RADS ≥ 2	163	7	0.96	0.78	0.86	0.83 (± 0.07)
	CO-RADS 3 + 4 + 5	169	37	0.82	0.86	0.84	0.78 (± 0.03)
	CO-RADS 4 + 5	102	52	0.65	0.84	0.73	0.75 (± 0.04)
	CO-RADS 5	72	82	0.47	0.95	0.63	0.75 (± 0.08)
Setting 5	CO-RADS ≥ 2	145	45	0.76	0.66	0.71	0.69 (± 0.07)
	CO-RADS 3 + 4 + 5	142	58	0.71	0.74	0.73	0.73 (± 0.06)
	CO-RADS 4 + 5	114	71	0.62	0.75	0.68	0.69 (± 0.06)
	CO-RADS 5	65	93	0.41	0.74	0.53	0.71 (± 0.08)
Setting 6	CO-RADS ≥ 2	156	21	0.88	0.70	0.79	0.76 (± 0.06)
	CO-RADS 3 + 4 + 5	160	49	0.77	0.84	0.80	0.77 (± 0.07)
	CO-RADS 4 + 5	120	43	0.74	0.79	0.76	0.79 (± 0.06)
	CO-RADS 5	66	68	0.49	0.75	0.59	0.75 (± 0.09)

### COVID-19 prediction

Here, we present the results for COVID-19 prediction (CO-RADS 5) for the best classifier in each setting. In setting 1, on the hold-out dataset, the random forest (RF) classifier classified patients with COVID-19 with an AUC of 0.88 ± 0.06. The sensitivity and specificity in identifying COVID-19 features for the RF algorithm was 0.86 ± 0.11 and 0.88 ± 0.06 respectively. After noise reduction, the performance of the RF model increased to AUC of 0.92 ± 0.06 with sensitivity = 0.91 ± 0.07 and specificity = 0.94 ± 0.04.

We observed a performance drop in the RF model for scenario 2, when trained on the Russian dataset and externally validated on the Italian dataset, and vice versa. The RF classifier achieved an AUC of 0.79 ± 0.07 without any noise reduction. The sensitivity and specificity of the RF model was 0.72 ± 0.10 and 0.73 ± 0.05 respectively. In setting 4, with noise reduction, AUC was 0.75 ± 0.08, sensitivity = 0.95 ± 0.03, and specificity = 0.63 ± 0.03 for COVID-19 prediction.

For setting 5 of scenario 2, the optimal model performance was observed with an AUC of 0.71 ± 0.06, sensitivity = 0.74 ± 0.09, and specificity = 0.66 ± 0.05. After noise reduction, the optimal model performance increased to 0.75 ± 0.09 (AUC), sensitivity = 0.75 ± 0.09, and specificity = 0.75 ± 0.05. The detailed evaluation metrics of the RF classifier in each setting are shown in Table 4.

### Interpretation of radiomic signature

**Fig. 4:**
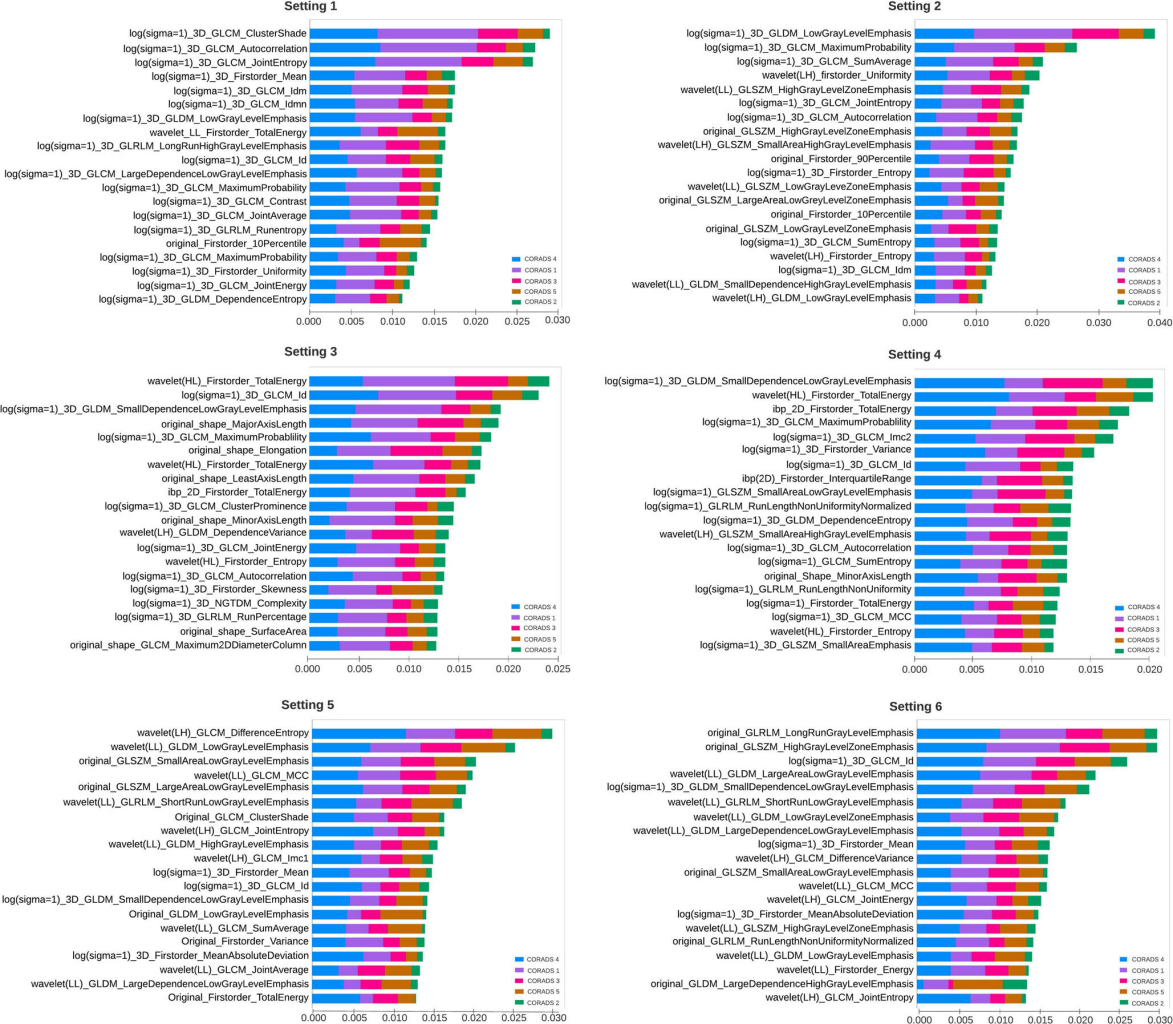
Top twenty feature visualization using SHAP for CO-RADS classification in each data setting. The features are arranged in a descending order of feature importance (SHAP values). Using this feature importance map, one can observe how each feature contributes to the machine learning model’s predictions and identifies the common features

To recognize the important features in each scenario for CO-RADS classification, we visualized the SHAP values of the top 20 features. The SHAP values of each setting are summarized in Fig. 4.

Out of the top 20 features responsible for CO-RADS classification of scenario 1, we found “log (sigma=1)-3D-GLCM Auto-correlation,” “log (sigma=1) 3D GLCM Joint Entropy,” “log (sigma=1) 3D GLCM Idm,” “log(sigma=1) 3D GLDM Low Gray Level Emphasis,” and “original Firstorder-10Percentile” features to be the most common in settings 1 and 2. Similarly, for other settings, important CO-RADS classification features can be realized in Fig. 4.

**Fig. 5:**
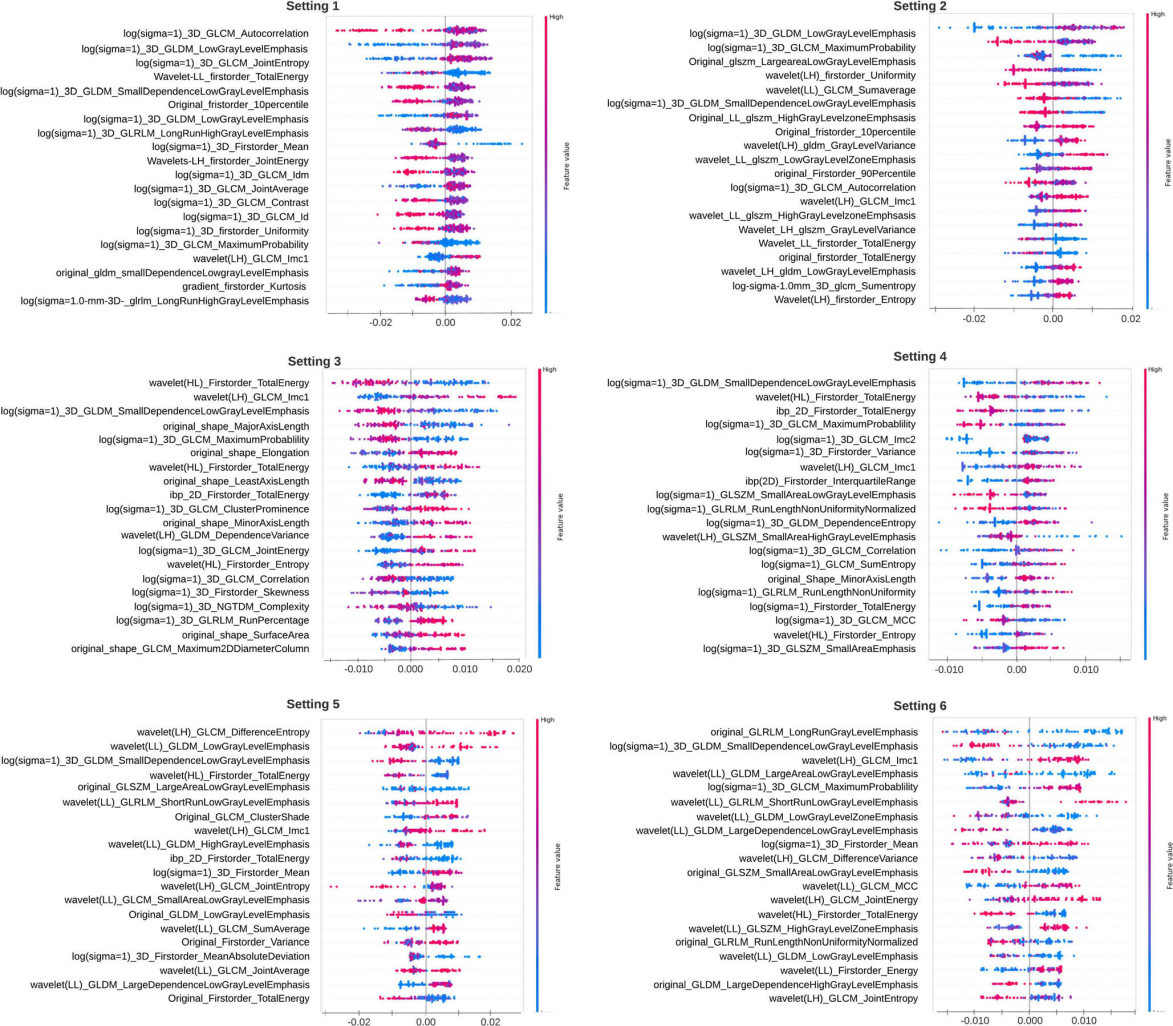
The class-specific feature summery plot for CO-RADS 5 using SHAP in each setting. The feature impact on the classification is observed by a positive SHAP value indicated by red color. For example, “wavelet (LH) GLCM Imc1” shows positive impact on CO-RADS 5 prediction in most of the settings

The individual summary plot showed class-specific features for CO-RADS 5 (Fig. 5). For each setting, a positive SHAP value indicates an increase in the risk of COVID-19. “Log (sigma=1) GLDM Small Dependence Low Gray Level Emphasis” showed negative impact, and “wavelet (LH) GLCM Imc1” showed positive impact on COVID prediction in almost all settings.

## Discussion

Several deep learning [28, 29], radiomic [30, 31], and integrated models [32, 33] have been developed since the outbreak of COVID-19, focusing on screening, diagnosis, and prognosis of COVID-19. To facilitate the translation of COVID-19 detection models into clinical practice, we followed a systematic approach to develop simple, generalizable, and reproducible automated COVID-19 detection models. In this study, (A) we successfully evaluated multi-site or public–private dataset scenarios and showed overall CO-RADS classification improvement by using noise reduction technique; (B) by choosing high-quality data from two clinical centers and heterogenous imaging protocols, we significantly reduced the population selection bias encountered in many recent studies as mentioned in a recent review [9]; (C) the annotation was done by three experienced radiologists from different countries avoiding the annotation bias that can occur when using inexperienced radiologists or radiologists from the same site; (D) by using a standardized annotation protocol, we made our study easy to implement and compare; (E) the interpretation of radiomic signatures via SHAP enabled us to pinpoint the key features that influence classification; and (F) finally, by carrying out validation procedures resembling the guidelines listed in category 2b and 3 of the Transparent Reporting of a multivariable prediction model for Individual Prognosis or Diagnosis (TRIPOD) statement, we have enhanced the quality of experimental design, and demonstrated the usefulness of the prediction models by testing the cross-dataset domain shift between different institutions [34].

Regardless of the type of validation scenario, CT radiomic features facilitated the identification of CT scans with suspected COVID-19 with accuracy of 84% (138/164) in a two-dimensional setting. Homayounieh et al, Fang et al, and Liu et al reported higher accuracies using a combined clinical–radiological radiomic signature, with their training based solely on a single demographic group, which may raise concerns of bias [30, 31, 35]. Lee et al used a deep learning algorithm and achieved accuracies similar to ours while investigating a heterogeneous population from more than eight countries [36].

We observed that among the two validation methods, the internal hold-out method had the highest AUC for COVID classification (CO-RADS 5) with 0.92 compared to the external validation (0.75). This shows that the model trained on multi-demographics and tested on internal hold-out performs better when compared to the model trained on single demographic data [36]. There have been many automatic models to differentiate COVID-19 from non-COVID-19 but a very few that have the capability to segregate comorbidities such as emphysema and lung cancer [2]. Notably, our multi-class classification model (RF) was able to identify other comorbidities with the highest accuracy of 85% (329/386). Prior works have shown that radiomics can be used to distinguish COVID-19 (CO-RADS 4) from other pneumonias (CO-RADS 3) on CT [37, 38]. We observed an average performance of 0.80 (AUC) in differentiating other community-acquired pneumonias (CO-RADS 3) in patients with suspected COVID-19, and in contrast to previous works, we used scans from patients with suspected COVID-19 without including scans from the general population.

Studies have shown the importance of using whole lung CT radiomics for predicting outcome and disease severity of COVID-19 patients compared with subjective radiologist assessment [30, 31, 39, 40]. Although the direct comparison of the features obtained in these studies cannot be compared to our study, we observed that textural features dominated feature importance for COVID-19 patients or CO-RADS 5 (Fig. 5). Interestingly, by employing noise reduction on non-contrast chest CTs, we were able to improve the performance for all the classifiers. This could be because apart from reducing noise and aiding dimensionality reduction of radiomic features, it helped in generalizing the scans acquired from different acquisition protocols (minimizing the variance).

Although the radiologists were from different countries (The Netherlands, Germany, and Italy), inter-observer agreement in CO-RADS scoring in multi-demographic data was substantial. Similar agreement was reported by Prokop et al, Lessmann et al, and Dilek et al [4, 7, 8].

Some of the evident limitations of this study are that our radiomic signature could also include epidemiological–laboratory parameters and clinical symptoms to provide a more comprehensive model. Secondly, it is a well-known fact that variation in the acquisition process, reconstruction parameters, or study protocol can result in different radiomic features. The degree to which this variation affects classification performance is an area of active research. The third limitation is that only non-contrast images were included. However, comparing and combining features engineered by deep learning approach would be the primary focus of our future work [41, 42].

## Conclusions

We have attempted to answer the question of whether we can use a systematic approach to construct a radiomic signature and employ simple ML classifiers that can effectively distinguish COVID-19 in a multi-demographic setting. The best classifier on average correctly designates the CO-RADS score in 80% of the cases. That is, by harnessing the power of radiomics combined with noise reduction, it is possible to predict the CO-RADS score from a non-contrast chest CT with a relatively high accuracy. Adopting the aforementioned model into clinical practice as a standardized tool may aid radiologists in classifying COVID-19. Lastly, this study design can be used as a research tool, facilitating reproducible and comparable models in the field of automated COVID-19 detection.

## Supplementary Information

ESM 1 (DOCX 18 kb)

## Abbreviations

AUC: Area under the receiver operation curve
CoLliAGe: Co-occurrence of local anisotropic gradient orientations
CO-RADS: COVID-19 Reporting and Data System
CT: Computed tomography
COVID-19: Coronavirus disease 2019
DLNR: Deep learning–based noise reduction
GLCM: Gray-level co-occurrence matrix
GLDM: Gray-level dependence matrix
GLRLM: Gray-level run length matrix
GLSZM: Gray-level size zone matrix
LBP2D: Local binary pattern 2D
LoG: Laplacian of Gaussian
LR: Logistic regression
MLP: Multilayer perceptron
NR: Noise reduction
RF: Random forest
RT-PCR: Reverse transcription–polymerase chain reaction
SHAP: SHapley Additive exPlanations

## Declarations

### Guarantor

The scientific guarantor of this publication is dr. ir. PMA (Peter) van Ooijen.

### Statistics and Biometry

Three of the authors have significant statistical expertise.

### Methodology

• Retrospective study

• Diagnostic study

• Multicenter study
