# Supplementary material for: Facilitating standardized COVID-19 suspicion prediction based on computed tomography radiomics in a multi-demographic setting
Source: Eur Radiol. 2022 Apr 1;32(9):6384–96. doi: 10.1007/s00330-022-08730-6 (PMC8973680; doi:10.1007/s00330-022-08730-6)
Supplement: Supplementary file 1 — (DOCX 18 kb) [file 330_2022_8730_MOESM1_ESM.docx]

# **Electronic Supplementary Material:**

## Multi-demographic readers

Reader 1 (GJ) works at the University medical center Groningen, the Netherlands and was a senior chest radiologist (>10 years of experience) with 4 months experience of reporting chest CTs of patients with suspected COVID-19. Reader 2 (MAF) was an interventional radiologist at the University medical hospital Heidelberg, Germany with >8 years of overall experience and 3 months experience in reporting chest CTs’ of patients with suspected COVID-19. Reader 3 (PG) is a chest radiology resident with 5 years' experience at the University Hospital in Rome, Italy and with 5 months experience in reporting COVID-19 chest CTs.

## Lobe segmentation

Before feature extraction, 3D lung lobe segmentation was carried out using an available lung segmentation algorithm [[24](bookmark://_bookmark23)]. After successful segmentation, each lobe was assigned a number between one to five and the voxels outside one of these numbered lobes were assigned zero. This helped to extract features from only the numbered candidate regions. The final preprocessing step included standard data augmentation for model training. The standard augmentation for lobe segmentation primarily involved resampling, resizing of each scan to 256 x 256 x 96, horizontal- vertical flip, rotation and histogram equalization.

## Feature engineering

After removal of highly correlated features from 1231 extracted radiomics features, using feature engineering that is PCC (p < 0.05) and applying ANOVA test, we obtained the following number of significant features for each scenario. For scenario-1, setting-1 (without noise reduction) feature engineering yielded 118 unique features. After noise reduction (setting-2) the number of features remained the same. However, for scenario-2, setting-3, the obtained 35 stable features increased to 47 features after noise reduction (setting-4). In setting-5, 109 pertinent features from the Italian datasets were extracted for CO-RADS classification. We observed no change in the number of features after noise reduction.

## Data and annotation access

The access for MosMed can be requested from here: https://mosmed.ai and CO-RADS annotations for the same will be made available on request to the corresponding author. Please refer to affiliations in the manuscript for contact details.
